# Supplementary material for: Human Brain Organoids: A New Model to Study Cryptococcus neoformans Neurotropism
Source: J Fungi (Basel). 2025 Jul 19;11(7):539. doi: 10.3390/jof11070539 (PMC12295756; doi:10.3390/jof11070539)
Supplement: Supplementary file 1 [file jof-11-00539-s001.zip › jof-3746254-supplementary.pdf]

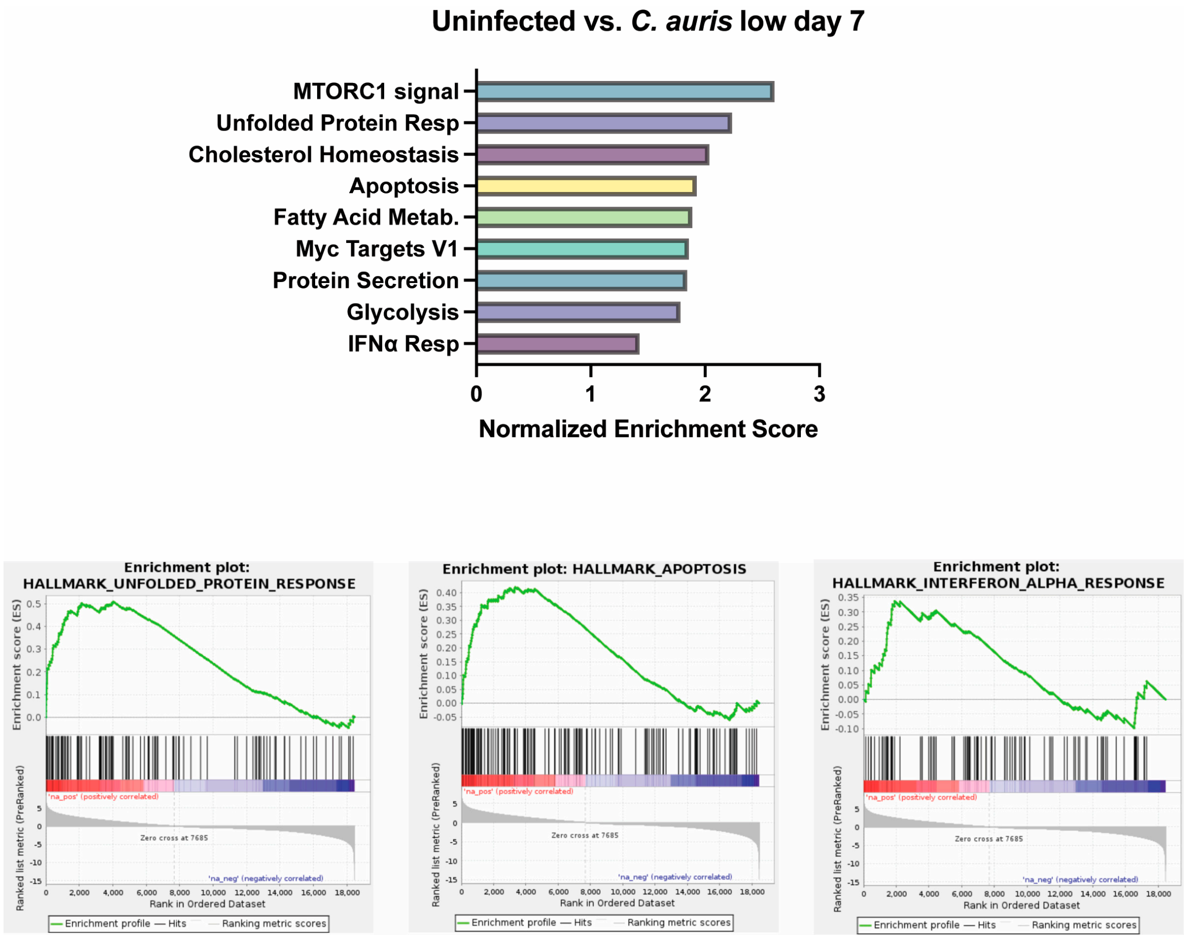

Figure S1. Gene set enrichment analysis of cerebral organoids following infection with *C. auris*: Graphed normalized enrichment scores of the hallmark pathways that were significantly enriched (FDR ≤ 0.05), and a set of enrichment plots for select pathways for organoids infected with *C. auris* seven days post-infection.
